# Reductions in root hydraulic conductivity in response to clay soil and treated waste water are related to PIPs down-regulation in *Citrus*

**DOI:** 10.1038/s41598-017-15762-2

**Published:** 2017-11-13

**Authors:** Indira Paudel, Shabtai Cohen, Lyudmila Shlizerman, Amit K. Jaiswal, Avi Shaviv, Avi Sadka

**Affiliations:** 1 Institute of Soil, Water and Environmental Sciences, ARO Volcani Center, Bet Dagan, 5025001 Israel; 2 Department of Soil and Water, The Robert H. Smith Faculty of Food Agriculture and Environment, The Hebrew University of Jerusalem, Rehovot, Israel; 3 Department of Fruit Trees Sciences, ARO Volcani Center, Bet Dagan, 5025001 Israel; 4 Institute of Plant Protection, ARO Volcani Center, Bet Dagan, 5025001 Israel; 5 Faculty of Civil and Environmental Engineering, Technion-Israel Institute of Technology, Haifa, 32000 Israel

## Abstract

Citrus hydraulic physiology and PIP transcript levels were characterized in heavy (clay) and light (sandy loam) soils with and without treated waste water (TWW) irrigation after a summer irrigation season and at the end of a winter rainy season recovery period. Consistent reductions in clay soils compared to sandy loam were found for fresh water (FW) and TWW irrigation, respectively, in root water uptake, as well as in hydraulic conductivity of whole plant (K_s_ plant), stem (K_s_ stem) and root (K_s_ root). Transcript levels of most PIPs down-regulated following TWW irrigation in both soils, but relative gene expression of three PIPs was significantly higher in summer for sandy soil and FW than for clay soil and TWW; their mRNA levels was significantly correlated to K_s_ root. A pot experiment, which compared short term influences of saline and TWW found that both treatments, compared to FW, reduced root water uptake and PIPs mRNA levels by 2-fold after 20 days, and the decreases continued with time until the end of the experiment. These latter data indicated that salinity had an important influence. Our results suggest that plant hydraulic adjustment to soil texture and water quality occurs rapidly, i.e. within days, and is modulated by PIPs expression.

## Introduction

Treated wastewater (TWW) is frequently used for irrigation in semi-arid and arid zones. In Israel, most fruit tree plantations, including citrus orchards, are irrigated with TWW of various qualities. Depending on the original water source and level of treatment, TWW might result in increased salinity, increased concentrations of organic and inorganic compounds, and increased levels of living organisms as compared to fresh water (FW)^1,2^, as well as changes in soil structure.

Soils can be highly variable in salt concentration, soil moisture, hydraulic properties, and nutrient availability^3^, and references within^4^. High concentrations of clay particles enhance soil compaction, reduce aeration and available soil water, and adversely affect growth^5,6^. Root growth is often slowed by a combination of soil physical stress and water quantity or quality. Stress may vary continually, depending on the location of the root in the soil profile, prevailing soil water conditions and soil texture^7^.

TWW contains high concentrations of saline components, and organic and inorganic suspended particles compared with fresh water (FW), which can lead to a breakdown in soil structure and reduced hydraulic conductivity, increased osmotic potential, decreased aeration and reduced root growth^8^. Reductions in root function and water uptake^4,7,9–12^, may be responsible for deceases in performance of plantations following TWW irrigation, as found for avocado, grapefruit, almond, peach and other fruit trees species^13–15^. Usually, the negative effects of TWW irrigation on yield are detected following long term exposure, 5 to 10 years^14,15^. Indeed, in a current field experiment, only mild reductions in productivity were detected after 2 years of exposure of mature trees to TWW (Paudel *et al*., In preparation). Plant hydraulic conductance is greatly affected by soil characteristics and water quality, especially in regards to salt concentration^16^. In leaves, hydraulic conductance (K_s_ leaf) is coordinated with leaf water potential^17^. In stems, hydraulic conductance is usually represented as stem specific conductivity (K_s_ stem), which is a function of vessel characteristics and embolism^18^. In roots, hydraulic conductance (K_s_ root) influences water uptake capacity, which depends on root surface area, root anatomy, and root water permeability^19–22^. The dominating driving force for water uptake is the water potential gradient, which depends on osmotic gradients^23,24^. While the effects of soil type and water quality on some physiological aspects have been studied extensively, the relationship between hydraulics and gene expression has received less attention.

Root permeability is thought to be regulated by Aquaporins^19,25^. Numerous studies have demonstrated the importance of aquaporins to plant water relations and plant hydraulics^20,26–28^. Reduced water uptake capacity in plants grown under abiotic stress, like drought and salinity, has been linked to a decrease in root hydraulic conductance^23,29,30^.

Aquaporin genes are usually divided into seven families, each consisting of multiple gene members: plasma membrane intrinsic proteins (PIPs), tonoplast intrinsic proteins (TIPs), nodulin-26-like intrinsic proteins (NIPs(, small intrinsic proteins (SIPs), GlpF-like intrinsic proteins (GIPs), hybrid intrinsic proteins (HIPs) and X-intrinsic proteins (XIPs)^31,32^. The question regarding their high redundancy was recently reviewed^33^. Among aquaporin, the PIPs constitute the largest number of genes, and are further divided into PIP1 and PIP2 subgroups according to their amino acid sequence similarity. The *Arabidopsis* genome contains five *PIP1* and eight *PIP2* genes, while the citrus genome contains four *PIP1* and four *PIP2* genes^34^. The reason for the large redundancy is yet to be identified; however, previous reports suggest that PIP1 and PIP2 are subjected to multiple modes of regulation, including protein phosphorylation, which affect their activity, trafficking and gating^35–40^. Moreover, co-expression of PIP1.1 proteins with an isoform from the PIP2 subfamily led to higher membrane permeability than that observed with the expression of a single PIP2 protein, suggesting that interactions between various isoform might affect activity^41,42^. Interactions of PIP with other proteins might also play a role in their regulation^35^.

The effect of environmental stimuli, such as, salinity, heavy metals, droughts, temperature and hypoxia on the expression and activity of aquaporins, mainly PIPs, is extensively described in the literature in many species including citrus^24,43–45^. A dominating role of aquaporin phosphorylation in governing K_s_ under various abiotic and nutritional stress conditions has been also described^46^. However, to the best of our knowledge, the effect of different soil types and irrigation with TWW on the expression of PIPs has not been reported.

We have recently described the effect of TWW irrigation in clay and sandy loam soils on growth, respiration and hydraulic conductivity of citrus root systems^8^. Root system functionality decreased following exposure to TWW, especially in combination with clay soil, indicating damage, and increased proline and decreased osmotic potentials indicated adaptive responses. Here, the effects on citrus whole plant, root, stem and leaf hydraulic conductivities are described, along with changes in the levels of PIP1 and PIP2 mRNAs. Measurements also included the recovery response after winter rainfall and fresh water irrigation. The results demonstrate the relationships and correlations between root hydraulic conductivity and the expression of three *CvPIP1/2* genes We show that although the effects on yield occur after years, significant and important effects on tree physiology, root hydraulics and PIP gene expression occur fast, i.e. within days, which, to the best of our knowledge, is a novel result.

## Results

### Photosynthesis, stomatal conductance, and transpiration

**Figure 1:**
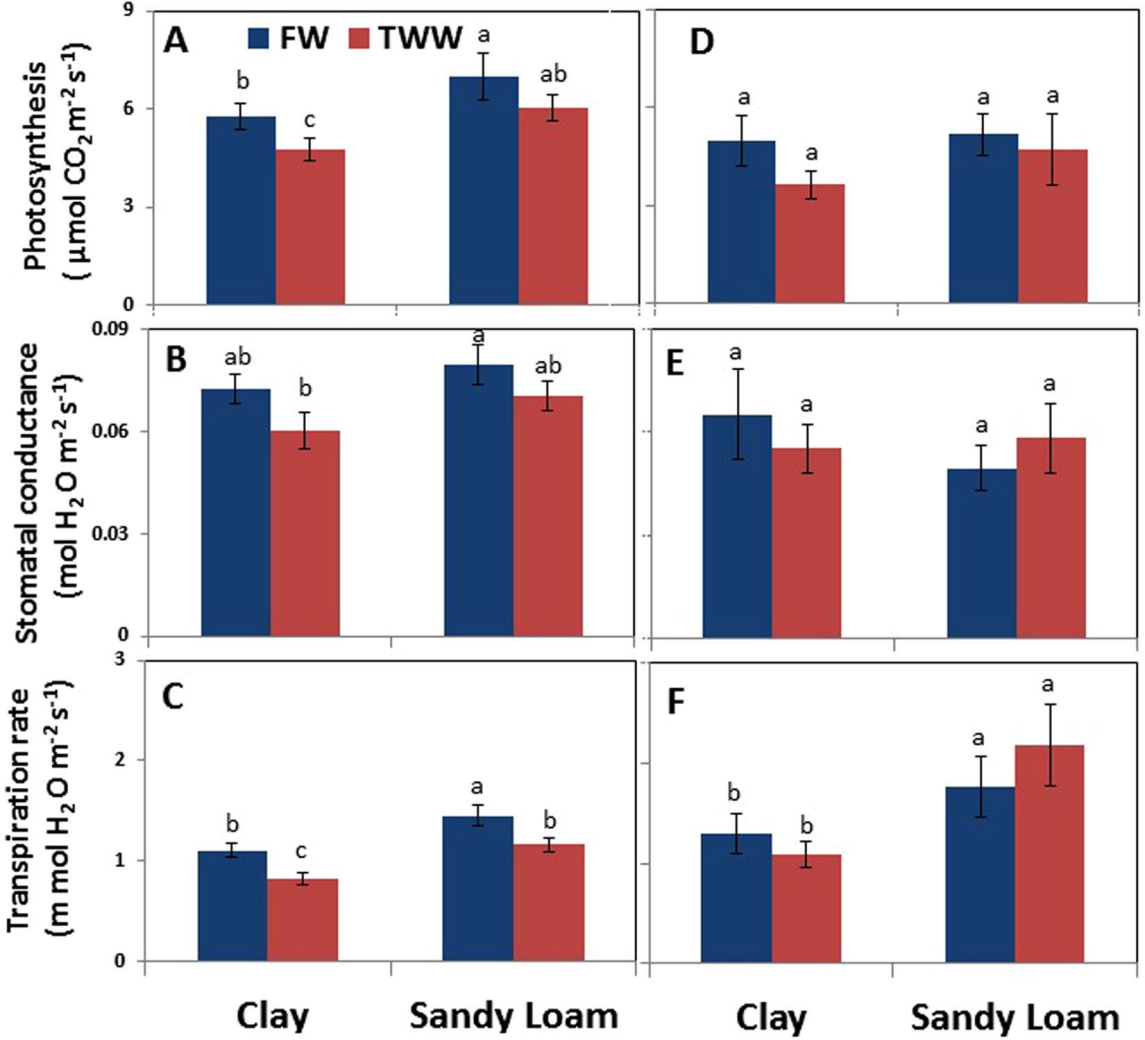
Effects of soil type and TWW irrigation on photosynthesis (**A**,**D**), stomatal conductance (**B**,**E**), and transpiration rate (**C**,**F**) on the days of root sampling for gene expression analysis in summer (**A**–**C**) and winter (**D**–**F**). New fully expanded leaves were used for measurements. Bars represent mean ± SE and different letters indicate significant differences (Tukey’s HSD test; P < 0.05, n = 4) between soil types and water qualities.

Leaf photosynthesis was significantly higher in sandy loam than in clay soil for both FW and TWW, by 18 and 25%, respectively (Fig. 1A). A significant ~19% reduction in leaf photosynthesis was found for TWW as compared to FW in clay, but not in sandy loam. There was no significant difference in photosynthesis rate in all tested conditions during the winter rainy season when rainwater and FW irrigation (when needed) leached the soil (Fig. 1D); hereafter referred to as the recovery period.

Overall, soil type and water quality did not significantly affect stomatal conductance (Fig. 1B). However, trees irrigated with FW grown in sandy loam had ~18% higher stomatal conductance (p < 0.01) than trees grown in clay soil irrigated with TWW. No significant differences were found in stomatal conductance during the recovery period (Fig. 1E).

In summer, transpiration rates in clay soil were 24% and 20% higher than in sandy loam for TWW and FW irrigation, respectively (Fig. 1C); and TWW resulted in 20% and 17% decreases in transpiration rate in clay and sandy loam, respectively. During the winter recovery period, trees grown in sandy loam had significantly higher transpiration rates (~30%) than trees grown in sandy loam with no significant differences between the water qualities (Fig. 1F).

### Leaf relative water content and water potential

**Figure 2:**
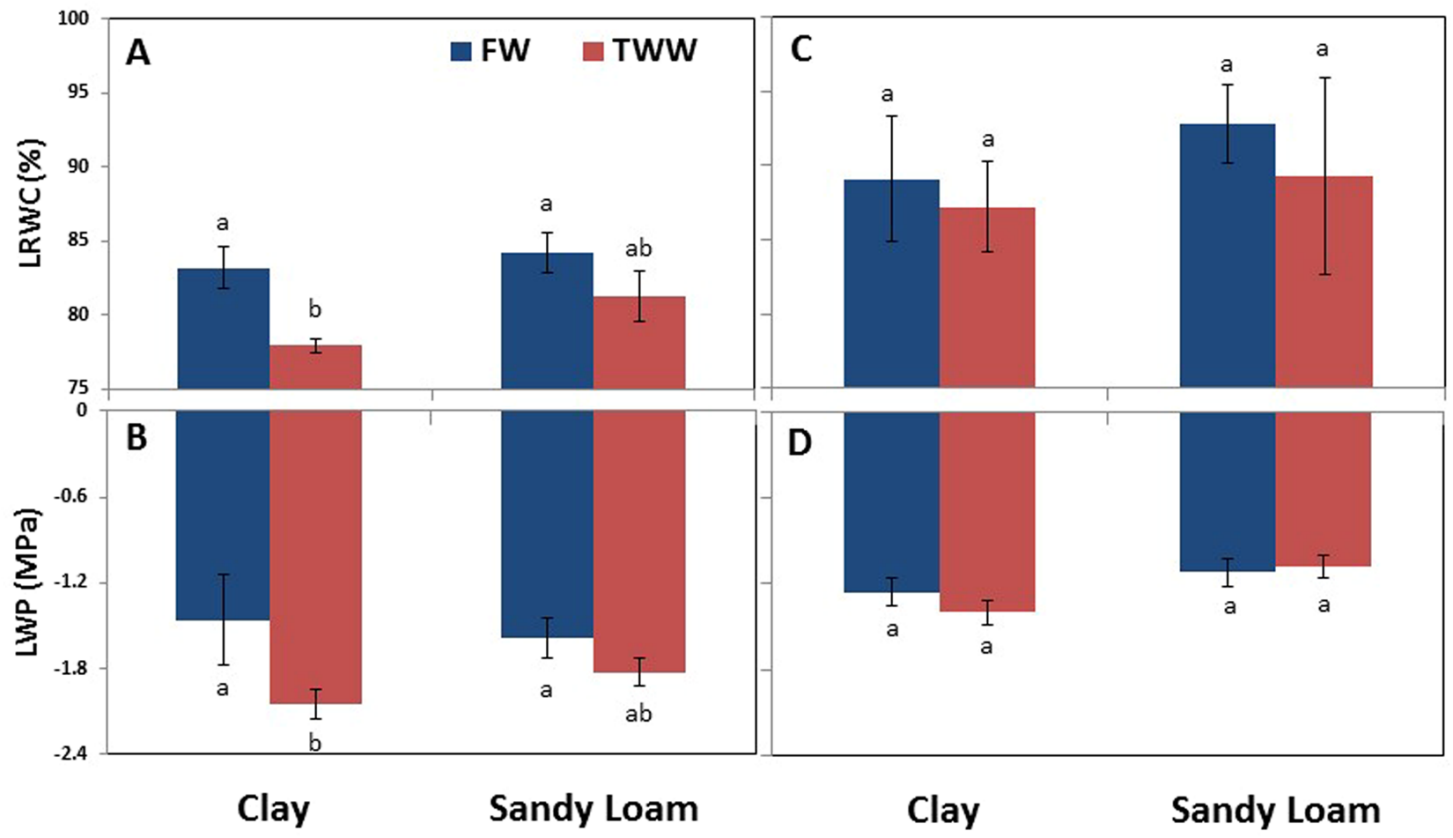
Effects of soil type and TWW irrigation on leaf relative water content (LRWC) (**A**,**C**) and water potentials (**B**,**D**) on the days of root sampling for gene expression analysis in summer (**A**,**B**) and winter (**C**,**D**). Bars represent mean ± SE and different letters indicate significant differences (Tukey’s HSD test; P < 0.05, n = 4) between soil types and water qualities.

Leaf relative water content (LRWC) was not affected by soil type in FW-irrigated trees (Fig. 2A). When plants were exposed to TWW, a significant decrease (13%) in LRWC was observed in clay soil but not in sandy loam. No significant difference was found between the various treatments during the recovery period (Fig. 2C).

Under FW irrigation, leaf water potentials were unaffected by soil type (Fig. 2B). Trees grown in clay soil irrigated with TWW showed a significant reduction (20%) in their LWP, as compared to trees irrigated with FW. No significant difference in LWP was recorded between the treatments during the recovery period (Fig. 2D).

### Root water uptake (Sap Flow) and whole plant hydraulic conductance

**Figure 3:**
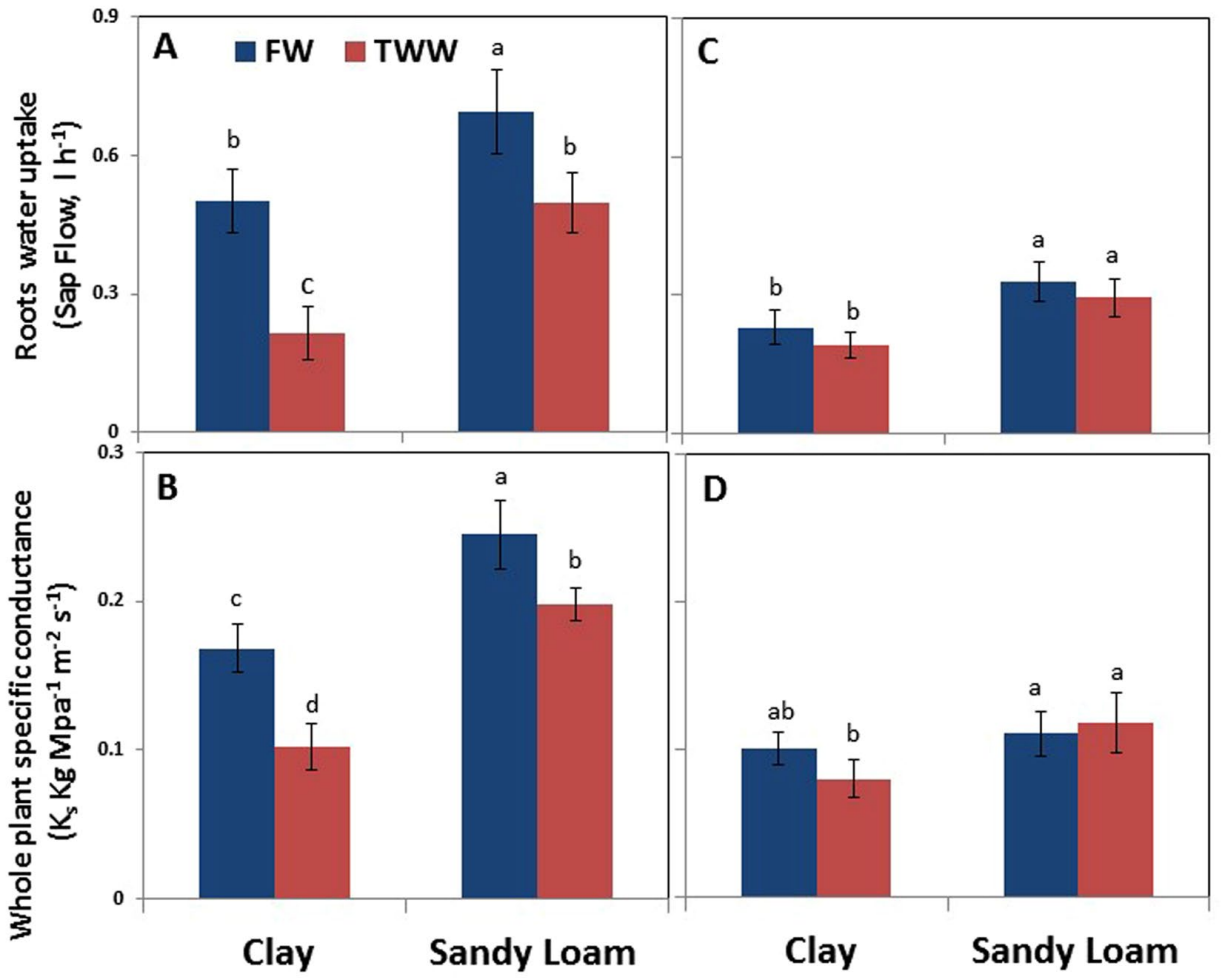
Effects of soil type and TWW irrigation on root water uptake (**A**,**C**) and whole plant hydraulic conductance (**B**,**D**) on the days of root sampling for gene expression analysis in summer (**A**,**B**) and winter (**C**,**D**). Bars represent mean ± SE and different letters indicate significant differences (Tukey’s HSD test; P < 0.05, n = 4) between soil types and water qualities.

Root water uptake was significantly higher in sandy loam than in clay soil under FW and TWW by about 28% and 53%, respectively (Fig. 3A). In both soil types, irrigation with TWW resulted in a reduction in root water uptake. However, while only a 20% reduction was detected in sandy loam, an over 2-fold reduction was detected in clay soil. During the recovery period, root water uptake of trees grown in sandy loam was significantly higher than in trees grown in clay soil (20%), with no apparent influence of water quality (Fig. 3C).

Soil type also affected whole plant specific hydraulic conductance (K_s_). For trees in sandy loam values were higher than for clay by about 22% for FW and 47% for TWW (Fig. 3B). TWW decreased K_s_ in both soils (35% in clay soil and 17% in sandy loam). A significant decrease was detected in K_s_ only in trees grown in clay soil irrigated with TWW as compared to trees grown in sandy loam, during the recovery period (~18%, Fig. 3D). Partitioning conductance into root-stem, and stem-leaf specific hydraulic conductance components (Supplementary Fig. S1) shows that both were equally affected by soil type and irrigation water quality.

### Leaf, stem and root specific hydraulic conductivity

**Figure 4:**
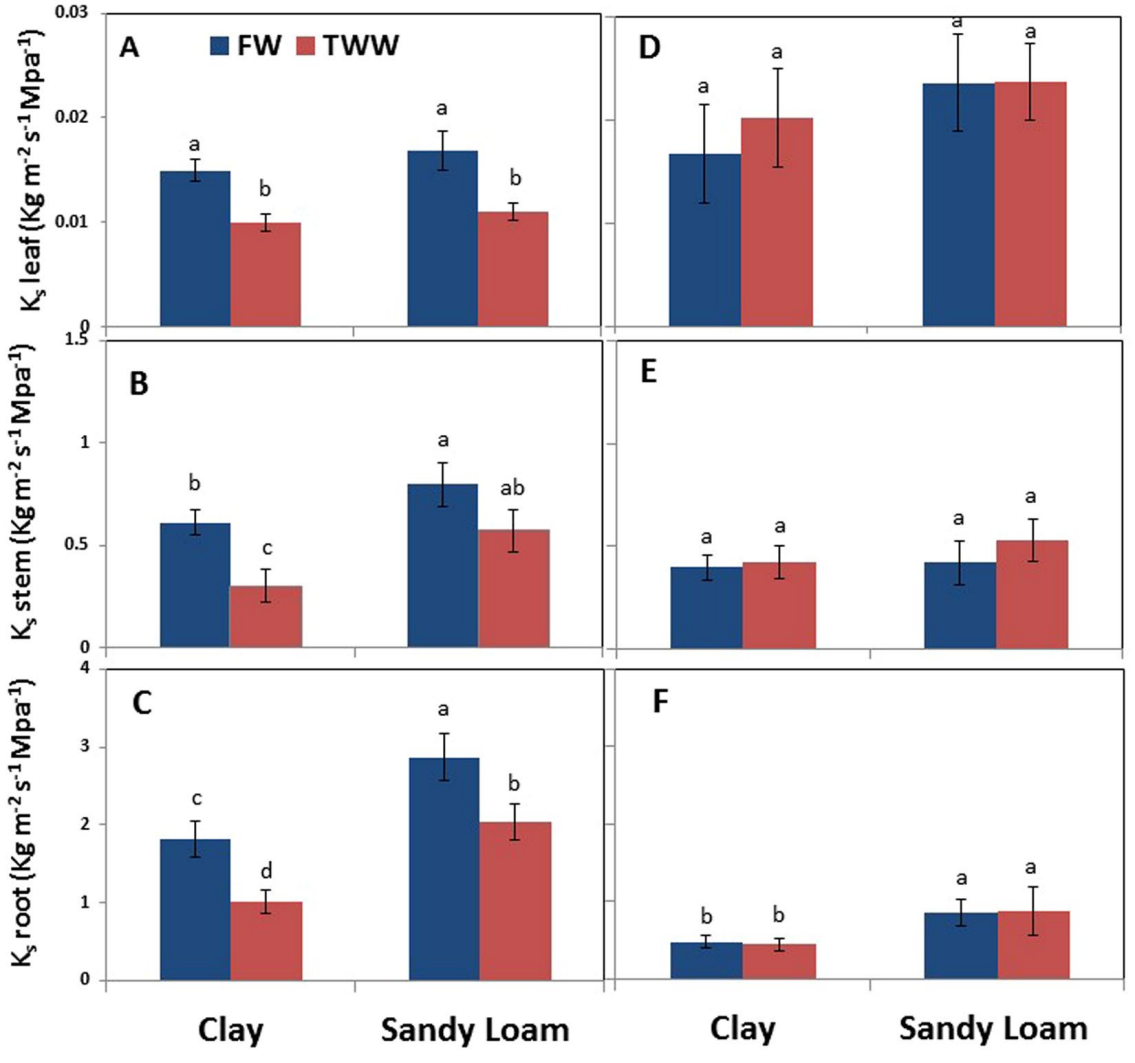
Effects of soil type and TWW irrigation on leaf (**A**,**C**), stem, and root specific hydraulic conductivity (**B**,**D**) on the days of root sampling for gene expression analysis in summer (**A**,**B**) and winter (**C**,**D**). Bars represent mean ± SE and different letters indicate significant differences (Tukey’s HSD test; P < 0.05, n = 4) between soil types and water qualities.

For FW, K_s,leaf_ was similar for both soil types (Fig. 4A), but K_s,stem_ and K_s,root_ were higher in sandy loam as compared to clay soil by about 25% and 35%, respectively (Fig. 4B and C). Significant reductions in K_s,leaf_, K_s,root_ and K_s,stem_ were detected in clay soil under TWW, as compared to FW by about 25%, 39% and 43%, respectively. In sandy loam, K_s,leaf_ and K_s,root_ were lower for TWW (23% and 25%, respectively) as compared to FW, but no significant reduction was detected in K_s,stem_. For TWW, K_s,stem_ and K_s,root_ were higher in sandy loam as compared to clay soil by 34% and 48%, respectively, but no difference was detected in K_s,leaf_ between the two soil types. As for most of the other parameters, no difference was detected in K_s,stem_ and K_s,leaf_ between the various treatments during the recovery period (Fig. 4D,E). However, K_s,root_ was lower in clay than in sandy loam for both water qualities (Fig. 4F).

### The mRNA levels of root PIPs is affected by soil type and water quality

**Figure 5:**
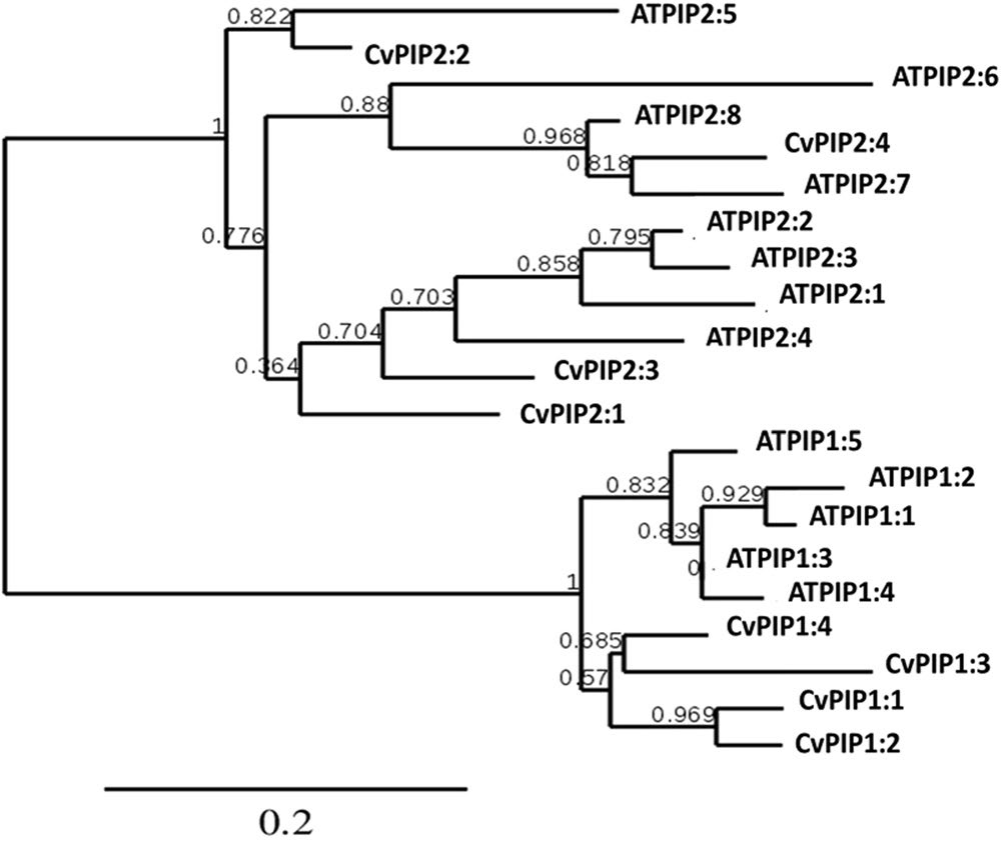
Phylogenetic tree of *Citrus sinensis* (Cv) PIP1 and PIP2 aquaporins along with *Arabidopsis thaliana* (AT) PIP1 and PIP2 aquaporins. The tree was produced using PhyML Software.

**Figure 6:**
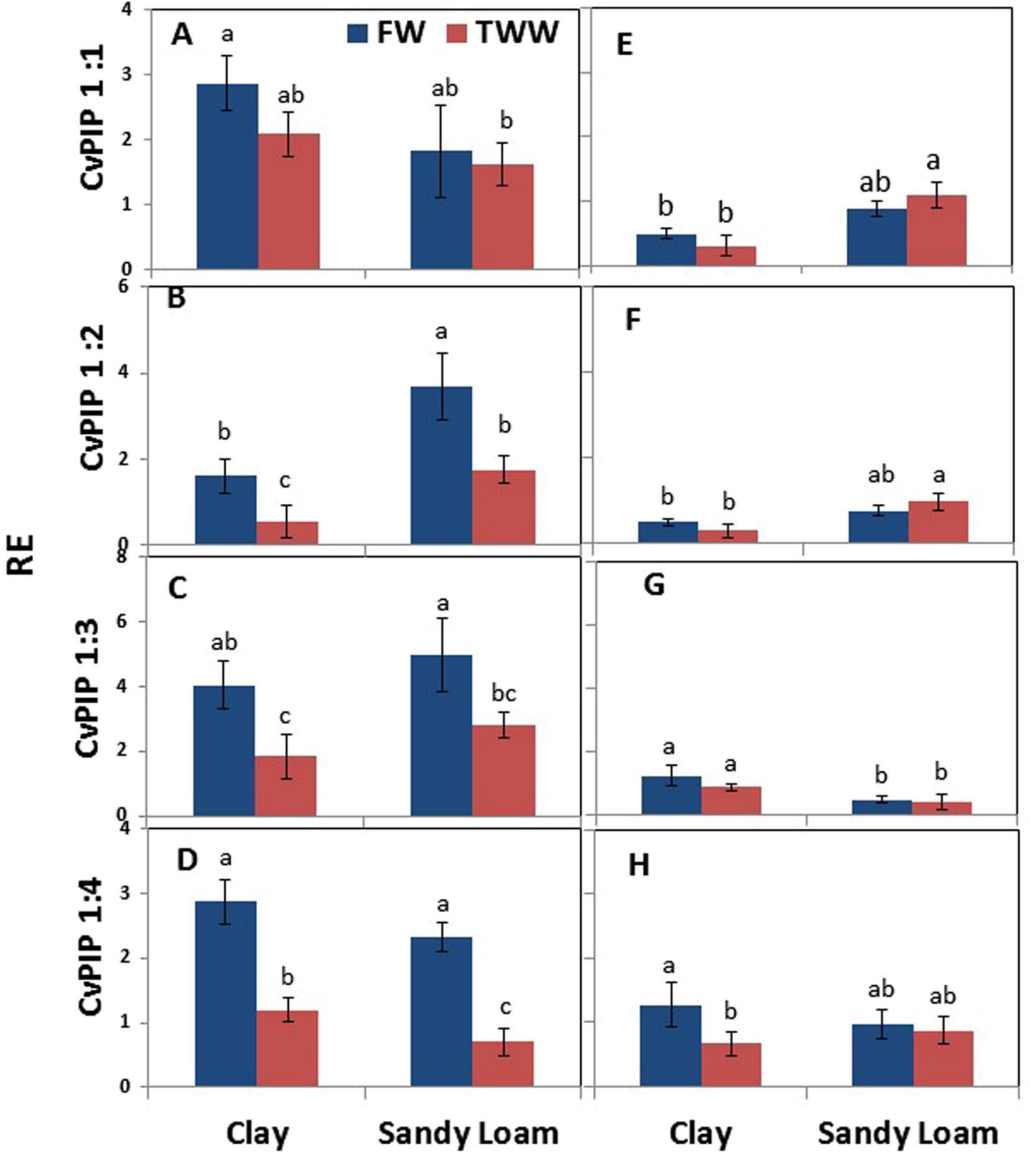
Effects of soil type and TWW irrigation on the expression level of root PIP 1 aquaporin genes in summer (**A**–**E**) and winter (**F**–**J**). Bars represent mean ± SE and different letters indicate significant differences (Tukey’s HSD test; P < 0.05, n = 4) between soil types and water qualities.

**Figure 7:**
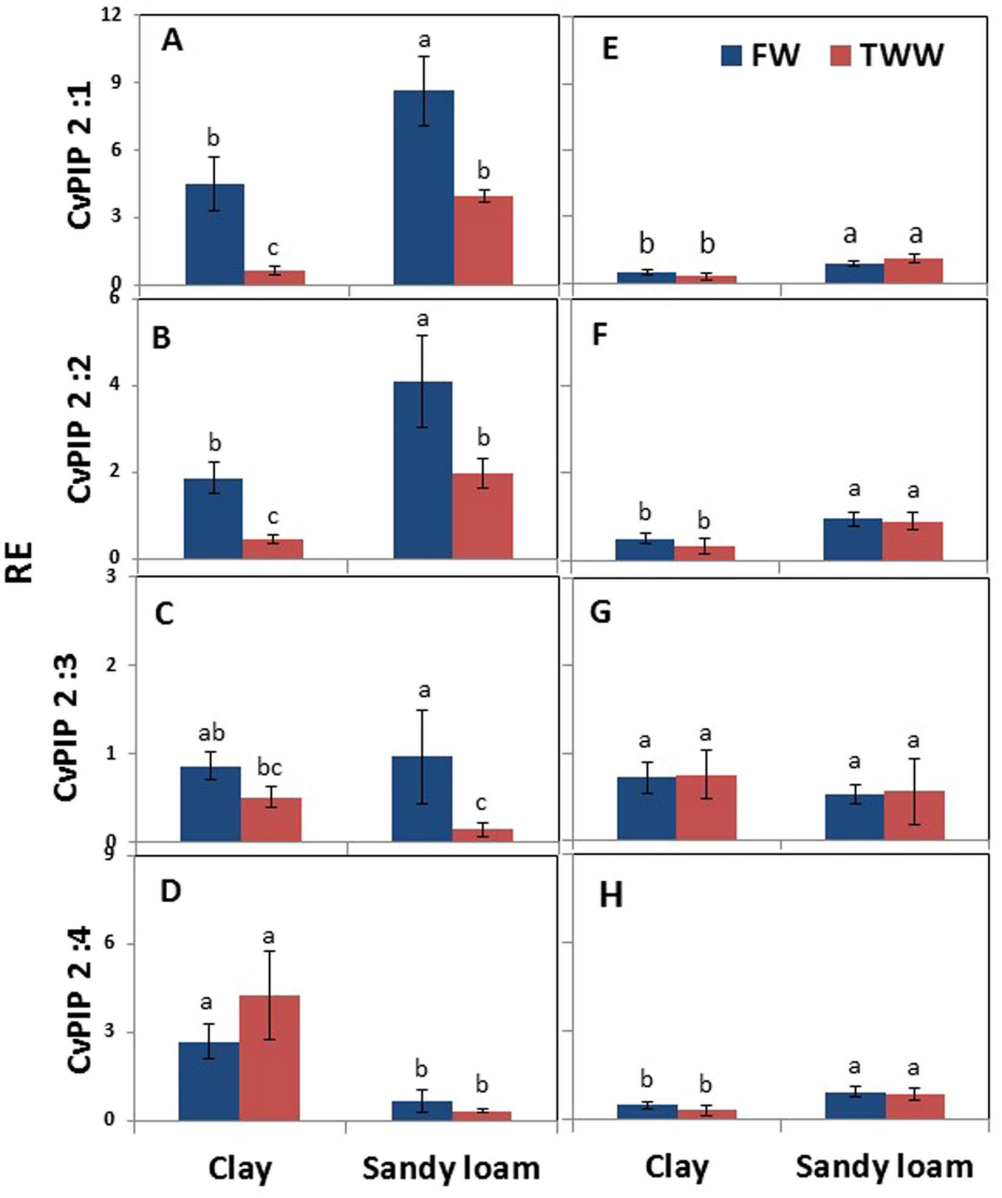
Effects of soil type and TWW irrigation on the expression level of root PIP 2 aquaporin genes in summer (**A**–**E**) and winter (**F**–**J**). Bars represent mean ± SE and different letters indicate significant differences (Tukey’s HSD test; P < 0.05, n = 4) between soil types and water qualities.

To identify *Citrus PIP* members, all PIP sequences were compiled from the *Citrus clementina* genome database (http://www.phytozome.net/) and from the *Citrus sinensis* genome database (http://citrus.hzau.edu.cn/orange/) by performing BLAST using *Arabidopsis* sequences as queries. Based on the two *Citrus* genomes, eight members of the PIP gene family were identified. Phylogenic analysis of the *Citrus* sequences along with their *Arabidopsis* counterparts (Fig. 5) revealed separation into two clear clades, four genes showing homology with the *Arabidopsis PIP1* gene family (named *CvPIP1:1–4*) and 4 genes showing homology with the *PIP2* gene family of *Arabidopsis* (named *CvPIP2:1–4*). The mRNA levels of the eight genes were analyzed in the roots by qPCR following two irrigation seasons (Figs 6A–D and 7A–D, for *CvPIP*1 group and for *CvPIP2* group, respectively), and following a 90 day recovery period of winter rainfall, which leached soil salts (Figs 6E–H and 7E–H, for *CvPIP1* group and for *CvPIP2* group, respectively).

Overall, significant changes in the mRNA levels of *CvPIP1* genes between the various treatments were relatively small, as compared to changes in the mRNA levels of *CvPIP2* genes (see below). The mRNA level of *CvPIP1:1* did not vary much between the treatments, and showed a 1.8-fold reduction only in trees grown in sandy loam irrigated with TWW as compared to trees grown in clay soil irrigated with FW. The mRNA level of *CvPIP1:2* was reduced 2.3 and 2.8-fold in TWW as compared to FW for clay soil and sandy loam, respectively. Transcript levels of PIP1:3 were reduced 2.2-fold and 1.8-fold in TWW as compared to FW in clay soil and sandy loam, respectively, and those of PIP1:4 were reduced 2.4-fold and 2.9-fold in TWW as compared to FW in clay soil and sandy loam, respectively.

mRNA levels of the four *CvPIP1* genes were usually reduced during the recovery period as compared to the irrigation period, with *CvPIP1:1*, *CvPIP1:2* and *CvPIP1:3* showing the largest reductions. As expected, no effect of water quality was detected on the mRNAs levels. mRNA levels of *CvPIP1:1* and *CvPIP1:2* were induced in sandy loam as compared to clay soil, those of *CvPIP1:3* were reduced in sandy loam as compared to clay soil, and transcript levels of *CvPIP1:4* were not altered by soil type.

Transcripts levels of *CvPIP2* genes seemed to be more affected by soil type than those of *CvPIP1* genes (Fig. 7), with the exception of *CvPIP2:3*, whose mRNA was similar in clay soil in FW under TWW, and also in sandy loam under FW (Fig. 7B). Trees grown in sandy loam irrigated with TWW had lower *CvPIP2:3* transcript levels than trees irrigated with FW, but the value did not vary significantly from that of trees grown in clay soil under TWW. The mRNA levels of *CvPIP2:1* and *CvPIP2:2* showed similar trends, 4 to 5-fold reductions in clay soil and TWW, as compared to FW, and about 2-fold decrease in sandy loam under TWW as compared to FW. Under FW, the mRNA levels of both genes was about 2 times higher in sandy loam than clay soil, and under TWW, about 5 times higher in sandy loam as compared to clay soil. Transcripts of *CvPIP2:4* displayed somewhat different patterns than those of *CvPIP2:1* and *CvPIP:2* genes. While the last showed clear induction in sandy loam as compared to clay soil under both TWW and FW, the mRNA level of *CvPIP2:4* was reduced in sandy loam as compared to clay soil under both water qualities, with 4-fold and 10-fold decreases between clay soil and sandy loam under FW and TWW, respectively. Also, while the transcript levels of *CvPIP2:1* and *CvPIP2:2* were reduced in clay soil under TWW as compared to FW, no such reduction was detected for *CvPIP2:4*.

The mRNA level of *CvPIP2* genes were significantly lower in winter during the recovery period (Fig. 6E–H), with the exception of *CvPIP2:3*, which showed no apparent reduction and differences between the various treatments. Transcript levels of the other three genes were significantly higher in sandy loam than clay soil, with similar values under both water qualities.

Three-way ANOVA (Table S1) showed that most parameters (Figs 1–7) changed significantly with season with the exception of stomatal conductance, Ks stem and transcript levels of PIP1:4 and PIP2:3. Season significantly interacted with soil type for Ks root and transcript levels of PIP1:1 and PIP2:4 and with water quality for Ks leaf, Ks root and the transcript levels of PIP1:2, PIP1:3, PIP2:1, PIP2:2 and PIP2:4. An additional significant three way interaction between season, soil type and water quality was found for transcript levels of PIP2:4.

### Relationships between root hydraulic conductivity and the mRNA levels of PIP1:2, PIP2:1 and PIP2:2

**Figure 8:**
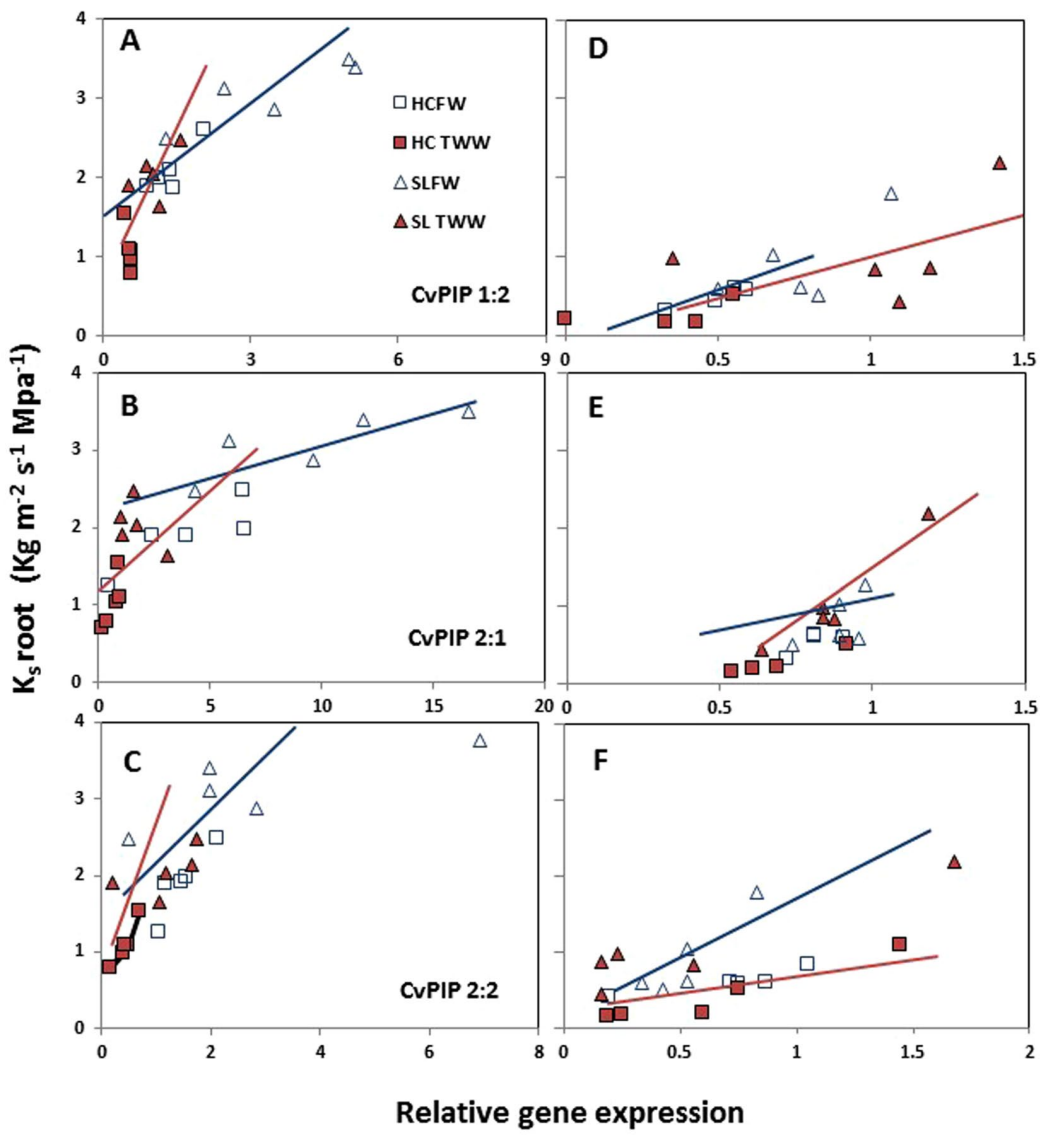
Relationships between root specific hydraulic conductivity and gene expression (**A** and **D**, CvPIP1.2; **B** and **E**, CvPIP2.1; **C** and **F**, CvPIP2:2) during summer (**A**–**C**) and winter (**D**–**F**) time, CFW- Clay soil FW, CTWW- Clay soil TWW, SLFW- Sandy Loam FW and SLTWW- Sandy Loam TWW. Values are for each individual replication (n = 4–5). The regression analysis for these three genes shows a significant relation between K_s_ root and PIPs relative expression (P < 0.01) in both soil types. See correlation value and P in Table S2.

Transcript levels of three genes, *CvPIP1:2*, *CvPIP2:1* and *CvPIP2:2*, were higher in sandy loam than in clay soil for both FW and TWW. Similar significant differences in these mRNA levels between soil types were also found during the recovery period. Therefore, correlation tests and regression analyses were performed for transcript levels and root hydraulic conductivity (Ks) of these genes for individual and combined water qualities for both soil types during differential irrigation and recovery periods (Table S3). Results of the correlation analyses for both types of soils are presented in Fig. 8A–C (differential irrigation period) and Fig. 8D–F (recovery period). Linear relationships were evident for all three genes with PIP1:2 in clay soil, and PIP2:1 in sandy loam showing the highest correlating values. Regression analyses showed that during differential irrigation period, all treatments (FW, TWW or combined irrigations) showed significance in clay soil (P < 0.05), while in sandy loam, FW treatment and combined irrigations were significant (Table S3). During the recovery period, in clay soil only the combined irrigation showed significance, while in sandy loam FW and the combined irrigation treatments were significant.

### Time period and factor response to root hydraulics and aquaporin’s expression

**Figure 9:**
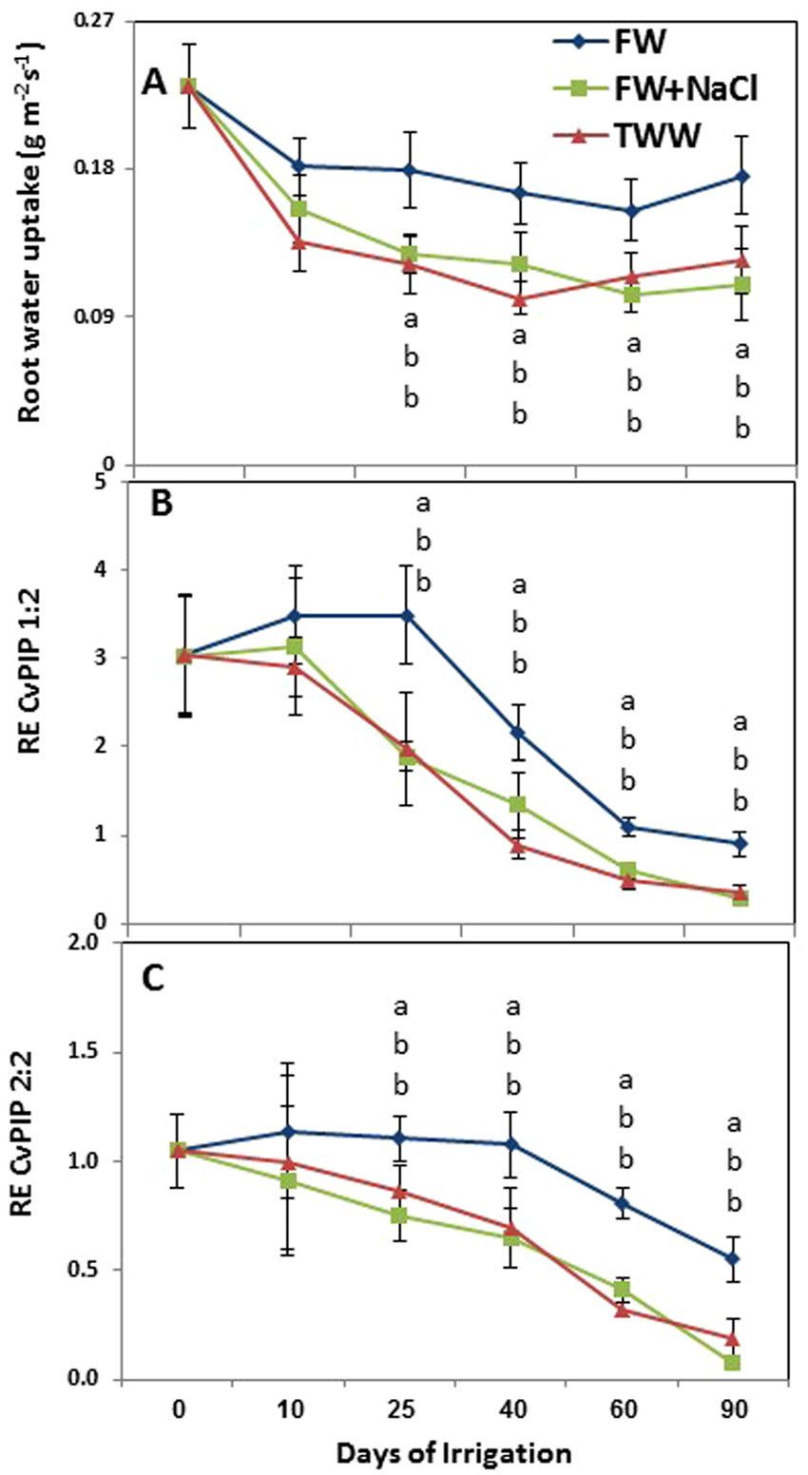
Time response of irrigation with TWW and FW with similar proportion of NaCl found in TWW (FW + NaCl) on root water uptake (**A**), relative gene expression of CvPIP 1:2 and CvPIP 2:2. Bars represent mean ± SE and different letters indicate significant different between water quality (One-way ANOVA; Tukey’s HSD test P = 0.05).

Most of *CvPIP* genes responded in a reduced transcript levels in TWW as compared to FW. Sodium chloride provides major component in treated waste water in Israel (Table S1). Therefore, we set on to examine time-dependent changes in root water uptake and in transcript levels of *CvPIP1:2*, and *CvPIP2:2* following exposure to three water qualities, FW, FW supplemented with NaCl (FW + NaCl) and TWW (Fig. 9).

Overall reduction in root water uptake per unit root surface area was detected within10 days under all treatments. However, after 25 days, root water uptakes were significantly lower in TWW and FW + NaCl as compared to FW, and differences were consistent until 90 days of irrigation treatments. Root water uptake was equally affected by TWW and FW + NaCl with insignificant periodic dynamics (Fig. 9A).

The mRNA levels of *CvPIP1:2* and *CvPIP2:2* also showed overall reduction under all treatments during experimental period (Fig. 9B and C). However, the reduction in FW was significantly smaller than in TWW and FW + NaCl, and it lagged behind that of the other treatments by 15 days for *CvPIP1:2* and 25 days for *CvPIP2:2*. Following 90 days of treatment, the mRNA level of *CvPIP1:2* and *CvPIP2:2* in FW was 3.2 and 2-fold lower, 6.5 and 5.2-fold lower than in TWW and in FW + NaCl was 5.5 and 5.8 fold lower to the 0 days of treatments, respectively. The relative expression of *CvPIP1:2* and *CvPIP 2:2* were 2.3 and 2-fold higher in FW than in FW + NaCl and TWW, respectively at 25 days and differences were increased to 3-fold, and 3 and 5- fold at 90 for TWW and FW + NaCl respectively.

## Discussion

As mentioned above, in the field, TWW effects on fruit trees performance and productivity become evident after years of exposure. Results presented here show that two irrigation seasons with the standard TWW quality used in Israel (with one recovery winter period in between) resulted in significant and important effects on tree physiology, hydraulics and the mRNA levels of most PIPs. Moreover, in the short term pot experiment, 20 days of exposure to TWW resulted also in significant differences in PIP transcripts levels. Considering the long term effects on tree productivity, these data, although indicating mild effects, are surprising, and to the best of our knowledge, are novel. Use of lower quality water may have resulted in a more dramatic effect, but our aim was to mimic, as much as possible, our local conditions.

Hydraulic conductance is fundamental to plant water relations; it can affect integrated responses such as stomatal conductance, photosynthesis and growth under changing environmental conditions^21^. The ability to maintain a high photosynthetic rate is considered one of the most important stress resistance characteristics of plants^47^. Here, photosynthesis and whole plant hydraulic conductance, K_s_, was reduced in clay soil and TWW but the reduction was minor in sandy loam soil (Fig. 1). Leaf relative water content (LRWC) and water potential (LWP) tend to vary similarly to K_s_^17^. In the present study, LWP were significantly lower by physical structure, LRWC was not but TWW reduced both in clay soil but not in sandy loam. It seems that sandy loam soil reduced osmotic and toxic stress by increasing leaching fraction and decreasing sorption^48^. Sap flow and whole plant specific conductance is represented by amount of water uptake by roots and used by plants, and controlled by root surface area, structure and size of root pore, radial and axial path ways, canopy volume and atmospheric demand, and also by formation of embolism^18^. In this study, when atmospheric evaporative demand was high, clay soil impeded root water uptake and whole plant hydraulic conductance was reduced, with further reduction for TWW (Fig. 4). Influences on root morphology, physiology, hydraulics and anatomy have been reported previously. On the other hand, except for leaves, significant differences in hydraulic conductivity were found for different soil types and water qualities in all the sections of the plant, and differences were greatest in roots (Fig. 4). This suggests that major limiting factors for water transport are in the roots as well as in the soil, and water quality significantly interacts with these, thus influencing tree water balance. The results support the previous study’s conclusion that in woody plants roots and stem contribute the major portions of whole plant hydraulic conductance^49^.

Water can move radially toward the xylem along three pathways: the apoplastic, symplastic, and transcellular pathways. The symplastic and transcellular pathways are collectively referred to as the ‘cell-to-cell’ pathway^19^. Previous studies have suggested that under drought and saline stresses, the cell-to-cell pathway plays an important role in water transport in the root and is driven by the osmotic gradient between the soil and root xylem sap^23^. In this study, the osmotic gradient was less affected by soil structure but the osmotic potential of root xylem sap was changed by TWW irrigation^8^. Thus, it is indicated that the osmotic driving force was not beneficial to water transport in citrus as observed in maize^44^. On other hand, it has been reported that the ‘cell-to-cell’ pathway can be largely controlled by the activity of aquaporin, which respond relatively rapidly and reversibly, causing changes in K_s_^50^.

Numerous studies have explored the variations in hydraulic conductivity and aquaporin expression in various plant species under normal and stress conditions, especially salt and drought^21,29,51^. To the best of our knowledge, the current work provides the first report of PIP mRNA levels for clay soil irrigated with low quality water, TWW. Overall, six out of eight analyzed genes responded to TWW in reduced transcript levels, usually regardless of soil type (Figs 6 and 7). Only PIP1;1 and PIP2;4 showed no response to TWW as compared to FW. Salinity is the major stress-imposing component of TWW (Supp. Table S1). Results of the second experiment (Fig. 9) demonstrated that time-dependent reduction in root water uptake and PIPs transcripts levels paralleled in TWW and in FW supplemented with salt, suggesting that salt indeed played a major role in the overall effect of TWW. Salt effect of root hydraulics and PIP activity was usually divided into short- and long-term effects (reviewed by^16^, which might explain some discrepancies found in the literature as to the effect of salt on PIP gene expression, with some works demonstrating reduced PIPs and hydraulic conductance, while other, including in citrus, showing no effect^20,26,44,52,53^. In the short term (a few hours following exposure to salt) plants respond to the osmotic shock and ionic effects of salt by reduced PIP activity, followed by reduced root hydraulics, most likely as an adaptive strategy to eliminate water loss from the roots, under conditions of low osmotic potential in the soil^16^. Following days of exposure to salt, there was an osmotic adjustment, release of ionic effect, induction in PIP activity and, at least partial recovery of hydraulics. It has been reported that the overexpression of PIPs genes results in rapid water uptake, which dilute the salt concentration^54^. Sutka *et al*.^21^ and Sade *et al*.^55^ concluded that enhancing the expression of aquaporin in the roots may provide an approach to compensate for the reduced soil water uptake under stress conditions. In line with this, following 20-days exposure of citrus seedlings to salt stress, all 8 PIP genes showed induction in their mRNA levels, suggesting long-term effects^34^. The data presented here also describes steady state levels following long-term exposure of the plants to TWW and heavy soil. However, even in the long term, root hydraulics and PIP mRNA levels do not recover to values of FW. Overall, this might suggest that TWW components other than salts, such as BOD and COD, might indeed affect PIP expression in root hydraulics in the long run.

Considering the combined effect of clay soil and TWW, the various PIP genes responded somewhat differentially, and they could be grouped into a few groups. Group 1 included PIP1:2, PIP2:1 and PIP2:2. Their mRNA levels were reduced under TWW as compared to FW and under clay soil as compared to sandy loam, with the lowest values under both TWW and clay soil. PIP1:3 transcript levels showed the same trend, but the differences between the two soil types were non-significant under both TWW and FW. It is suggested that these PIPs are highly operative under normal growth conditions and reductions in their expressions under TWW and clay soil were consistent with the previous studies showing that PIPs transcripts level decreased under stress conditions, encouraging cellular water conservation by reducing membrane water permeability and limiting loss of cellular water^21,26^. Moreover, our data also demonstrated positive relationships, both by correlation test and regression analysis, between transcript levels of group 1 and root hydraulic conductivity. Others also reported good correlations between the expression of various PIPs and root hydraulic conductance^21,56–58^, suggesting their importance in water uptake, especially under beneficial conditions. During recovery period, when water availability and quality were not limiting, the expression of group 1 PIPs was greatly reduced, but still, the expression in sandy loam was higher than in clay soil, suggesting that constraining growth effects imposed by clay soil were mitigated, but only to a certain extend. Similar growth effects were observed in walnut with the induction in the mRNA levels of PIP2:1 and PIP2:2, and in rice^59^, Zea mays^60^ and other plants^25^. Group 2 included only PIP2:4; its transcript levels were remarkably induced in clay soil as compared to sandy loam, but no differences were detected between the two water qualities in both soil type. It might be assumed that this protein plays an important role in the adaptation to inferior soil-related irrigation, but not directly to water quality^30,40,61–63^. During recovery period, transcript levels in clay soil were greatly reduced, even below that of sandy loam, suggesting that under FW, the effect of the clay was mitigated. Group 3 included PIP1:4 and PIP2:3, their transcripts levels were similar in both soil types under FW, but under TWW, they were higher in clay soil as compared to sandy loam. It is suggested that, similarly to Group 1 genes, these PIPs are mostly operative under relatively high water quality. Interestingly, unlike other PIPs, transcripts levels of Group 3 PIPs during recover period was not altered significantly, as compared to irrigation period, suggesting their importance for water uptake round the year. Group 4 included only PIP1:1, its mRNA levels did not vary between the various treatments, pointing up for its importance for water uptake under all conditions. During recovery period, its mRNA levels were reduced, especially in clay soil, suggesting that constrains resulting from soil type were mitigated. Overall, results of this study show correlations between PIP1 and PIP2 mRNA levels and changes in hydraulic conductivity imposed by the effect of TWW, clay soil and the combination of the two. Therefore, results support the hypothesis that the regulation of root and whole plant hydraulics in different soil textures and water qualities might be largely regulated by aquaporin, PIP1 and PIP2.

## Material and Methods

### Experiment I: Plant material and growth conditions

The experiment was conducted during 3 years (2013 to 2015) at the Technion Israel Institute of Technology (Technion City, Haifa, Israel) on saplings (2.5 years old) of Rio Red grapefruit (*Citrus paradise Macfadyen*) grafted on Volkameriana (Citrus volkameriana Volkamer) rootstock. Trees were grown in lysimeters, which were constructed of plastic barrels 1.2 m deep and 0.5 m in diameter with suitable drainage at the bottom. Sandy loam (Hamra) and clay (Grumosol) soils were used, both with a history of long term TWW irrigation, as described elsewhere^64^. Irrigation treatments were applied after transplanting one sapling in each of 24 lysimeters^8^. Two water qualities (Fresh water, FW, and treated waste water, TWW) were used as treatments in both soils with four replications of each treatment in four randomized blocks^8^. Each lysimeter with a single tree was considered as a replication, and lysimeters were arranged in blocks. Drip irrigation was applied and excessive drainage was collected from an outlet pipe at the bottom of each lysimeter. Irrigation for clay soil was 7 L Tree^−1^ and 9 L Tree^−1^ for sandy soil, applied once every three days during the summer irrigation season. The experiment continued for 90 days (December, January and February) in winter, to check the possibility of recovery of activity following leaching of soil by rainfall (<650 mm per rainfall season) and supplementary fresh water irrigation when needed. Measurements of soil chemistry after winter leaching were performed at the end of the winter rainfall season in the second week of March.

### Photosynthesis rate, stomatal conductance, and transpiration rate

Leaf photosynthesis, stomatal conductance, and transpiration were measured with a portable photosynthesis system (Li-6400XT; LI-COR Inc., Lincoln, NE, USA) between 9:00 am and 11:00 am simultaneously with root sampling (see below). Fully expanded leaves were measured at a photon flux density of 1000 mmol m^−2^s ^−1^. The flow rate through the chamber was 500 mmol m^−2^s ^−1^ and the leaf temperature was 25 °C. Four leaves were measured for each treatment.

### Leaf relative water content and water potential

Leaf relative water content (LRWC) was measured as described by Paudel *et al*.^65^. Fully expanded leaves were weighed immediately after being picked to determine the fresh weight (FrWt). Leaves were floated in distilled water for 6 h, dried with filter paper and weighed to determine the turgid weight (TW). The discs were then dried at 60 °C in a forced-air oven for 78 h to determine the dry weight (DW). The relative water content was calculated as follows,

1 $$LRWC=\frac{FrWt-DW}{TW-DW}\ast 100 \% $$

The water potential of the fully expanded leaves was measured as pre-dawn at 5.00–6.00 am and for leaf - between 10:00 am and 12:00 am using a pressure chamber (ARIMAD 2, MRC Ltd., Holon, Israel). Each treatment included six replications.

### Root water uptake (Sap Flow) and whole plant hydraulic conductance (K_plant_)

Root water uptake was measured with thermal dissipation sap flow sensors installed in the trunk base (for details see^66^. K_plant_ per unit leaf area was calculated based on Cohen *et al*.^67^ as,

2 $${K}_{plant}=\frac{Sap\,flow}{{\phi }_{soil}-{\phi }_{leaf}}$$

Leaf area per sapling was determined from the number of leaves counted on each and from 50 leaves per sapling, which were harvested and scanned to determine the area of an average leaf.

Pre-dawn leaf water potential was assumed to correspond to soil water potential, ${\phi }_{soil}$.

### Root, stem and leaf specific hydraulic conductivity

Root, stem and leaf samples were collected on the same days and times when other measurements were conducted. Root and stem branches were wrapped in wet cotton clothes inside black plastic and put on ice during transport to the laboratory. Leaf samples were wrapped only in aluminum foil and in the plastic and delivered in ice to the laboratory.

Branch sections (roots/stem) were recut under degassed water in the lab to lengths of 10 cm (stem) and 5–7 cm (roots) to avoid excessive branches, and potential artifacts related to cavitation^68^. K_s_ was measured at a hydrostatic pressure of 0.007 MPa by forcing water from a 0.7 m water column in burettes as described by Paudel *et al*.^8^.

Fresh weight of leaf samples was measured immediately following arrival to the laboratory, after which samples were dipped in distilled water. Leaf hydraulic conductivity was measured using the leaf rehydration kinetics method described by Brodribb *et al*.^17^:

3 $${K}_{leaf}={C}_{bulk}\,\mathrm{ln}[\frac{{{\rm{\Psi }}}_{o}}{{{\rm{\Psi }}}_{f}}]/t$$

Where C_bulk_ is leaf capacitance, Ψ_0_ is initial water potential, Ψ_f_ is final water potential after rehydration and t is time since rehydration began. C_bulk_ was estimated according to Blackman *et al*.^69^.

### Experiment II

Time-dependent changes in hydraulics and aquaporin PIPs mRNA levels were examined in a separate experiment using seedlings of 8 month old Volkameriana rootstocks (*C. volcameriana* Volckmer) grown in 2 L container filled with sandy loam soil. Irrigation treatments included FW, TWW (similar to the lysimeter experiment) and a treatment where NaCl was added to reach similar salinity level as TWW (Table S1), FW + NaCl. Root water uptake, fine root surface area, and aquaporin PIPs mRNA levels were followed from the beginning of the experiment for 90 days.

### Determining mRNA levels of aquaporins

Total RNA was extracted from approximately 2 g of fine roots using the CTAB extraction method^70^. RNA was treated with RQ1 RNase-free DNase (Promega, Fitchburg, WI) according to the manufacturer’s instructions. RNA quantity was analyzed in a NanoDrop ND-1000 Spectrophotometer (Wilmington, DE) and RNA quality was determined by Agilent Bioanalyzer (Santa Clara, CA). cDNA was synthesized from 1 μg RNA using OligoT as a primer and M-MLV Reverse transcriptase (Fermentas, Burlington,Ontario, Canada) in a final volume of 25 μl containing the commercially supplied buffer. Primers were designed based on genomic and EST sequences (Phytozome, http://www.phytozome.net/, HarvEST, http://harvest.ucr.edu/) using Primer 3 software (Table S2). Real-time PCR was carried out in a reaction mix containing 2 mM gene-specific forward and reverse primers, 3μl cDNA (diluted 1:16), KAPA SYBR FAST qPCR Master Mix^71^ Universal (KAPA Biosystems, Boston, MA), and Ultra-Pure water (Fisher Biotech, Wembley, Australia) in a final volume of 12 μl in a Corbett Rotor-Gene 6000 (Qiagen, Venlo, The Netherlands). Reactions were run for 40 cycles of 10 s at 95 °C, 15 s at the annealing temperature for each gene, 20 s extension at 72 °C, and the threshold level was determined. For the dual-labeled probe reactions, real-time PCR was carried out in a reaction mix containing 2 mM gene-specific forward and reverse primers, 2.5 mM dual-labeled probes, 3 μl cDNA (diluted 1:16), TaqMan Universal PCR^71^ Master Mix (Applied Biosystems, Inc., Foster City, CA) and Ultra-Pure water in a final volume of 12 μl in the Rotor-Gene 6000. Reactions were run for 40 cycles of12 s at 95 °C, 60 s annealing and extension at 60 °C, and the threshold level was determined. Standard curves were generated for each gene using serial cDNA dilutions. Relative concentration of the product was calculated by the algorithm of the Rotor-Gene software using the CT value. Relative expression (RE) was defined as the ratio between the relative concentration of each gene and that of β-actin.

### Statistical analysis

All statistical analysis were carried out using SAS (JMP) software (SAS Institute, http://www.sas.com). TWO-Way ANOVA was carried out both for soil type and water quality and Tukey’s HSD was used for mean separation when ANOVA results were significant. Fitting X and Y were done for correlation tests and ANOVA was run to test significance.

## Electronic supplementary material

Supplementary File
