# Supplementary material for: Reductions in root hydraulic conductivity in response to clay soil and treated waste water are related to PIPs down-regulation in Citrus
Source: Sci Rep. 2017 Nov 13;7:15429. doi: 10.1038/s41598-017-15762-2 (PMC5684345; doi:10.1038/s41598-017-15762-2)
Supplement: Supplementary file 1 — Supplementary File [file 41598_2017_15762_MOESM1_ESM.pdf]

Reductions in root hydraulic conductivity in response to clay soil and treated waste water are related to PIPs down-regulation in *Citrus*

Indira Paudel, Shabtai Cohen, Lyudmila Shlizerman, Amit K. Jaiswal, Avi Shaviv and Avi Sadka

Figure S1: Stem-leaf (A, C) and Root-stem (B, D) specific conductance on the days of root sampling for gene expression analysis in summer (A, B) and winter (C, D). Bars represent mean ± SE and different letters indicate significant differences (Tukey's HSD test; $P<0.05$, n= 4) between soil types and water qualities. These values are components of whole plant specific conductance presented in Fig 3, calculated from eq. 2 using φ_{stem} from measurements of mid-day stem water potential[69].

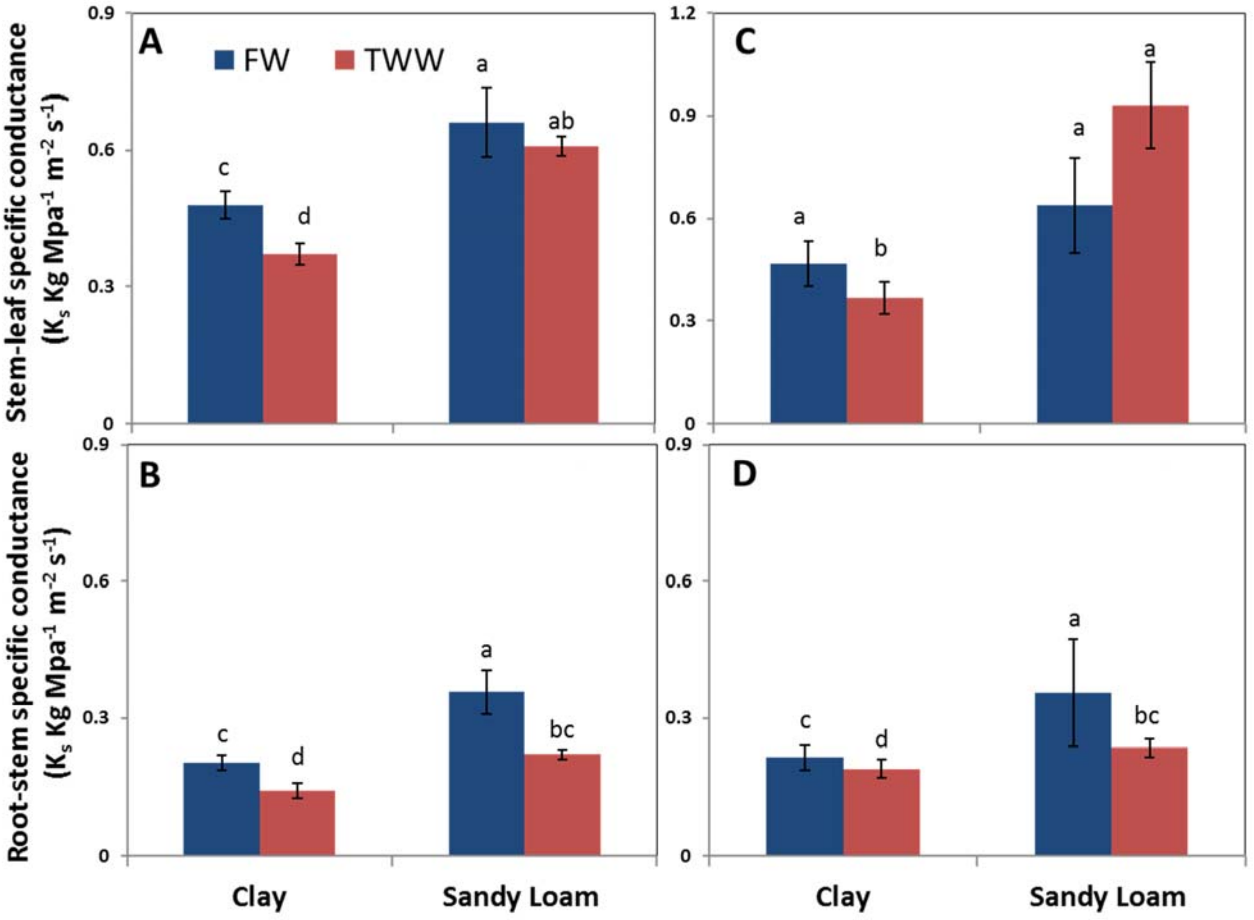

Table S1. Three-Way ANOVA (P-values) for season (summer/irrigation and winter/recovery), and its interaction with soil type and water quality (WQ) for all the measured parameters (Fig 1 to 7)

Parameter	Season	Season* Soil Type	Season * WQ	Season*Soil type*WQ
Physiology				
Photosynthesis	**0.002**	0.09	0.87	0.76
Stomotal conductance	0.056	0.2	0.78	0.56
Transpiration	**0.0001**	0.12	0.62	0.67
LRWC	**0.0001**	0.22	0.56	0.34
LWP	**0.001**	0.3	0.56	0.45
Hydraulics				
Root water uptake	**0.001**	0.12	0.45	0.78
Plant hydraulic conductance	**0.0023**	0.22	0.35	0.56
Ks leaf	**0.002**	0.1	**0.02**	0.45
Ks stem	0.51	0.2	0.5	0.78
Ks root	**0.0003**	**0.051**	**0.02**	0.12
mRNA expression				
CvPIP1:1	**0.004**	**0.03**	0.42	0.72
CvPIP1:2	**0.028**	0.37	**0.026**	0.17
CvPIP1:3	**0.032**	0.4	**0.039**	0.19
CvPIP1:4	0.091	0.72	0.26	0.57
CvPIP2:1	**0.0011**	0.25	**0.03**	0.67
CvPIP2:2	**0.01**	0.34	**0.03**	0.41
CvPIP2:3	0.85	0.8	0.19	0.38
CvPIP2:4	**0.002**	**0.03**	**0.0013**	**0.013**

Table S2. Correlation test (R^2) and regression analysis (P-value) of three CTPIP genes and root specific hydraulic conductivity (Fig 8) which were significantly affected by both soil type and water quality in both time periods. Correlation test and regression analysis were presented for individual and combined water quality for different soil type and seasons.

**Season**	**Soil type**	**WQ**	**R^2**	**P-value**	**R^2**	**P-value**	**R^2**	**P-value**
			**PIP 1:2**		**PIP 2:1**		**PIP 2:2**	
Summer	HC	FW	0.77	**0.038**	0.77	**0.042**	0.82	**0.03**
		TWW	0.89	**0.014**	0.69	0.058	0.92	**0.01**
		Combined	0.78	**0.0007**	0.61	**0.008**	0.71	**0.002**
	SL	FW	0.63	**0.041**	0.68	**0.04**	0.69	**0.045**
		TWW	0.28	0.17	0.37	0.098	0.45	0.07
		Combined	0.35	**0.049**	0.75	**0.001**	0.58	**0.01**
Winter recovery	HC	FW	0.49	0.13	0.71	**0.045**	0.85	**0.02**
		TWW	0.59	0.07	0.67	0.052	0.92	**0.009**
		Combined	0.67	**0.012**	0.75	**0.005**	0.84	**0.0002**
	SL	FW	0.55	**0.045**	0.37	0.3	0.86	**0.02**
		TWW	0.17	0.5	0.94	**0.005**	0.89	**0.01**
		Combined	0.32	**0.098**	0.72	**0.0019**	0.74	**0.001**

Table S3. Physiochemical properties of applied irrigation water in the pot experiments (values are mean± SE for 90 days. The measurement units are mg/l except for pH and EC (ds/m).

WQ	TSS	COD	BOD	N	P	K	pH	EC	Na	Cl
FW	7.3 ± 0.7	13 ± 0.7	5.4 ± 0.7	82 ± 7	33 ± 3	84 ± 5	7.3 ± 0.02	0.71 ± 0.04	45 ± 7	45 ± 20
FW+NaCl	7 ± 0.8	14 ± 0.8	5.5 ± 0.5	87 ± 6	31 ± 2	79 ± 10	7.8 ± 0.08	1.85 ± 0.06	190 ± 20	310 ± 45
TWW	30 ± 2	110 ± 26	17.3 ± 3	89 ± 9	38± 4	87 ± 6	7.77 ± 0.03	1.9 ± 0.05	188 ± 23	412 ± 35

Table S4. Primers used for citrus CvPIP aquaporin reference gene qPCR analyses

Gene ID	Gene	Primer	Product size(bp)
	β ACTIN	F-CAATGGCCCCAACCTTAGC<br>R-GTCTGGTCCATCCATTGTCCA	
orange1.1g018895m	CvPIP1:1	F-TGATCAGAGCCATTCCCTTC<br>R-ACACAGAAGCAGGTCCAGGT	164bp
orange1.1g023021m	CvPIP1:2	F-TCGCTGTGTTCTTGGTTCAC<br>R-CTGGTGGTACAAAGCTGCAA	173bp
orange1.1g023069m	CvPIP1:3	F-AGGATTCACACGGAGCCACCT<br>R-TGCTTTTGGATTTGGACACG	156bp
orange1. 1g023107m	CvPIP1:4	F-TCACAGGAACTGGCATCAAC<br>R-TTAAGCCCTGGACTTGAAGG	
orange1.1g023108m	CvPIP2:1	F-GCCACTGATCCCAAGAGAAA<br>R-CCAAGCCTTGTCCTTGTTGT	174bp
orange1.1g019681m	CvPIP2:2	F-TGTGTTCATGGTTCACTTGG<br>R-TGAATGGTCCAACCCAGAAG	137bp
orange1.1g022966m	CvPIP2:3	F-CCATTCATTGGAGCTTTCGT<br>R-GGCCTTCGTAACAGACTTGC	174bp
orange1.1g023370m	CvPIP2:4	F-TGAACCACCAACCAGAATAGC<br>R-AATGATTCACGGAGACAAAGC	162bp
